# An exploration of changes in cognitive and emotional empathy among medical students in the Caribbean

**DOI:** 10.5116/ijme.5412.e641

**Published:** 2014-09-24

**Authors:** Farid F. Youssef, Paula Nunes, Bidyadhar Sa, Stella Williams

**Affiliations:** Faculty of Medical Sciences, University of the West Indies, St. Augustine, Trinidad and Tobago, West Indies

**Keywords:** Medical students, Caribbean, cognitive empathy, affective empathy

## Abstract

**Objectives:**

This study explored the empathy profile of students across five years of medical training. In addition the study examined whether the Jefferson Scale for Physician Empathy correlated with a measure of cognitive empathy, the Reading the Mind in the Eyes Test and a measure of affective empathy, the Toronto Empathy Questionnaire.

**Methods:**

The study was a comparative cross-sectional design at one Caribbean medical school. Students were contacted in class, participation was voluntary and empathy was assessed using all three instruments Descriptive statistics were calculated and differences between groups evaluated using non-parametric tests.

**Results:**

Overall 669 students participated (response rate, 67%). There was a significant correlation between the Jefferson Scale of Physician Empathy and the Toronto Empathy Questionnaire (P = 0.48), both scales indicating a decline in medical student empathy scores over time. There was, however, little correlation between scores from the Reading the Mind in the Eyes Test and the Jefferson Scale of Physician Empathy. Female students demonstrated significantly higher scores on all three measures.

**Conclusions:**

Medical students’ lower empathy scores during their final years of training appear to be due to a change in the affective component of empathy. These findings may reflect an adaptive neurobiological response to the stressors associated with encountering new clinical situations. Attention should be paid not only to providing empathy training for students but also to teaching strategies for improved cognitive processing capacity when they are encountering new and challenging circumstances.

## Introduction

The past decade has seen a renewed call for a focus on professionalism in medical education. Today, professionalism is understood to be the foundation of the doctor-patient relationship with emphasis being placed on clinical empathy, a competence considered core to the professional attributes of doctors, critical to their communication skills and consequently, one of the hallmarks of patient centred care. Empathy impacts on clinical outcome. Indeed, a recent retrospective study that examined over 20,000 patients with diabetes concluded that higher physician empathy resulted in reduced metabolic complications..^1^ Not only is this study the largest of its kind, but it also supports previous work that demonstrated higher physician empathy is linked to better glycaemic control, reduced duration of the common cold and greater patient satisfaction and empowerment.^2^-^4^

The importance of empathy in clinical practice has been recognized since the early twentieth century when Olser first advocated professional empathy as the way “to find out” the emotional state of patients.^5^ Until that time physicians had been encouraged to share their understanding with patients but not their emotions, a type of unemotional involvement which resulted in ‘detached concern’. Today such a lack of physician engagement is considered inadequate for mutual physician-patient satisfaction^6^-^8^ and clinical empathy is now characterized as having two major components, one affective and the other cognitive. Whereas the cognitive component of empathy is the intellectual ability to understand the patient’s perspective and to view the world from that perspective, the affective component describes the capacity to imagine and to enter into the experience and feelings of the patient.^9^

With this renewed emphasis on empathy in medical practice has come a focused attention on the teaching and evaluation of empathy during medical training. Accordingly, several scales have been designed and developed to measure it. In particular, the Jefferson Scale of Physician Empathy Student Version (JSPE-S) developed in 2001 has been widely used in North America and Europe where studies have noted a decline in student empathy occurring across training and even during residency. A worrying aspect of this trend is that this decline is greatest during the third year when students embark upon their clinical training and begin interfacing with patients.^10^^, ^11

Factor analysis of the JSPE-S has identified ‘perspective taking’ as responsible for the largest proportion of variance when using this measurement tool. ‘Perspective taking’ is defined as the tendency to spontaneously adopt the views of the other person and, as such, the JSPE-S can be considered a measure of cognitive empathy.^12^ However, as already noted empathy has both cognitive and emotional components such that any attempt to measure changes in empathy should seek to consider both of these components.

It is also worth noting that most measures currently used are self-reporting scales which have their own limitations, including social desirability bias. Evidence from neurobiology is now providing insights into the underlying neurological mechanisms responsible for empathy and this has been coupled with attempts to develop more psychophysiological and behavioural measures thereof.^9^^, ^13 Such measures should theoretically be more objective and less susceptible to the individual bias associated with self-report tools. One test that is gaining acceptance is the Reading the Mind in the Eyes Test (RMET) which was first developed to detect ‘subtle cognitive dysfunction’ in normal adults. This test, an emotional recognition task, is also considered a measure of cognitive empathy.^14^

Given that previous work using the JSPE-S has demonstrated a decline in empathy among our students between the beginning and end of year one^15^ this study seeks to expand upon these findings. We re-administered the JSPE-S across all five years of study and compared it with an affective measure of empathy, the Toronto Empathy Questionnaire (TEQ) and a cognitive measure, the RMET. This study should help us to better understand the multidimensional nature of empathy in our population and provide evidence to support curricular changes and other strategies directed towards helping medical students become more caring health professionals. The aims were therefore to (i) conduct a cross-sectional study of empathy levels across all five years of training in a medical school, (ii) measure both cognitive and affective components of empathy across five years of training and (iii) determine whether there is any correlation between the three scales of empathy (JSPE-S, RMET and TEQ) in their measurement of its cognitive and affective components. 

## Methods

### Participants and setting

Participants were drawn from the population of medical students attending the Faculty of Medical Sciences (FMS) at the University of the West Indies, St. Augustine, Trinidad and Tobago. This population comprises a rich ethnic mix having been previously colonised by the Dutch, Portuguese, French and British using African slaves and East Indian indentured labour. These two latter groups comprise the vast majority of the population. As a result, this twin-island state in the Caribbean is today a heterogeneous ‘melting pot’ of ethnicities, cultures and religions, evidenced by fourteen national holidays celebrating various religious and cultural festivals. The University of the West Indies opened the medical school in Trinidad and Tobago, at the St. Augustine Campus in 1989 and now boasts an annual intake of approximately 300 medical students. Most of these students enter university directly from secondary school and follow a five year curriculum in which they begin the transition to clinical studies in year three.

### Study design

This research project was designed as a comparative cross sectional study among medical students across all five years within the Faculty of Medical Sciences. Ethical approval was granted by the Faculty’s Ethics Committee. Data were collected between November 2012 and January 2013.

### Instruments

Students were asked to complete three instruments: the Jefferson Scale of Physician Empathy Student Version, the Toronto Empathy Questionnaire and the Reading the Mind in the Eyes Test.

Student participation was voluntary; no course credits or rewards were provided. Other information collected included specialization preference as well as demographic information, age, gender and ethnicity.

The JSPE-S is a self-reporting measurement tool of empathy which has been widely validated at medical schools in several different languages in more than 30 nations. It has demonstrated excellent validity and test-re-test reliability.^16^^, ^17 We have also previously successfully administered this instrument at our institution.^15^ This instrument consists of twenty.^20^ questions measured on a seven-point Likert scale ranging from 1=strongly disagree to 7= strongly agree, with 4=not sure. Answers to all questions were summed to give a total score out of 140, with higher scores indicative of a more empathetic tendency among students.

The second instrument completed was the Toronto Empathy Questionnaire (TEQ). The TEQ is a recently developed self-report tool that conceives of empathy as a primarily emotional process. It consists of sixteen (16) questions that are designed to measure the affective component of empathy. Examples of questions used within the TEQ include, “I become irritated when someone cries”; “When I see someone being treated unfairly, I do not feel very much pity for them” and “I enjoy making other people feel better”. All questions are scored on a five-point Likert scale ranging from 0=never to 4=always. Similar to the JSPE-S, individual responses are summed to give a total score out of 64 with higher scores providing evidence of higher levels of affective empathy. The TEQ has demonstrated good internal validity and test-retest reliability.^18^ While not designed specifically for use within the medical profession, the scale was validated on college students who are of a similar age profile as the students we assessed in this study. The third instrument completed, the Reading the Mind in the Eyes Test (RMET), is considered a measure of cognitive empathy. This test consists of 36 photographs that reveal only the eye region of the individuals depicted. Participants are required to identify the emotion being expressed and select an answer from four options that are provided and placed at the four corners of the photograph. One is given for each correct answer; no response is considered as evidence that the emotion was not identified. The maximum possible score is 36 with mean scores typically ranging from 24-30. This instrument has shown good validity and reliability demonstrating test-re-test reliability up to one year later.^19^^, ^20 It has also been previously used in medical student populations.^21^

### Sampling

Using convenience sampling the three selected instruments were administered to medical students in the 2012-2013 academic year. Overall 669 students participated in this study (response rate of 67% given total enrolment is approximately 1000 students). The response rate per year and other demographic information are summarized in Table 1 Approximately twice as many females participated in the study compared to males (65% vs 35%) but this is consistent with the demographic profile of the current FMS student population.

### Procedure

Students were contacted in class and provided verbal informed consent before beginning the study. Identifying information such as names or identification numbers was optional. Students were given unlimited time to complete the instruments.

Data were analysed in SPSS v17. Descriptive information was calculated for each scale including the mean, standard deviation and median. Given that the data collected were ordinal, comparisons between scales were performed using the non-parametric tests, Kruskal-Wallis test (K-W) and Spearman’s correlation (ρ). Statistical significance was set at 0.05.

**Table 1.** Demographic characteristics of students (N = 669)

Variables	N (%)	Response rate (%)
Age		
<22	337 (50)	
22 – 27	298 (45)	
>27	28 (4)	
Gender		
Male	231 (35)	
Female	438 (65)	
Ethnicity		
African	142 (21)	
Indian	381 (57)	
Mixed	120 (18)	
Other	12 (2)	
Specialization		
People-Oriented	285 (43)	
Technology-Oriented	212 (32)	
Undecided	169 (25)	
Cohort		
Year 1	224 (34)	87
Year 2	136 (20)	62
Year 3	102 (15)	53
Year 4	94 (14)	51
Year 5	113 (17)	62

## Results

### Jefferson Scale of Physician Empathy

**Figure 1.**
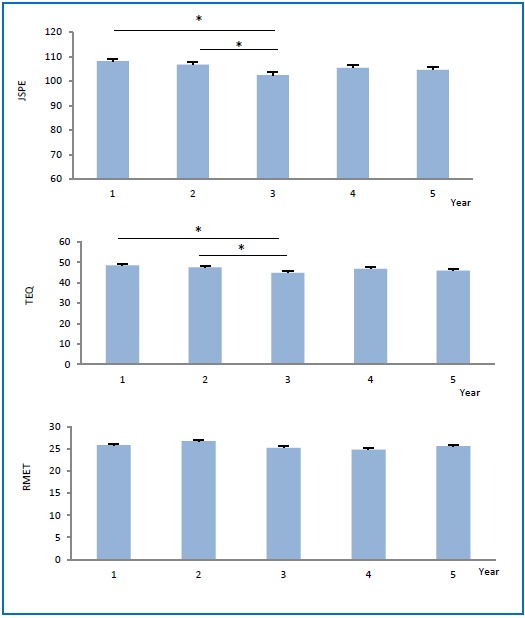
Change in empathy scores over the five years of medical school

Six hundred and sixty seven (667) persons successfully completed the JSPE-S. Descriptive statistics are presented in Table 2 Cronbach’s alpha was 0.77 and suggested moderate reliability. Overall there was a significant decreasing trend in mean empathy scores (X^2^(4)=19.62, p<0.001) from years one to three with a small increase in years four and five, Figure 1

**Figure 1** Change in empathy scores over the five years of medical school

Post hoc analysis demonstrated a significant difference between mean empathy scores in year 1 and year 3 (X^2^(1)=15.42, p<0.001) and year 2 and year 3 (X^2^(1)=7.01, p=0.008). There was a medium effect size, Cohen’s d = 0.5 and there were no other significant post hoc interactions, p>0.05. Females mean empathy scores were significantly higher than males (X^2^(1)=10.38, p<0.001) but there were no significant effects for age, ethnicity and specialization, table 2 Both males and females demonstrated a similar decline in empathy scores though the effect size in males was greater (Cohen’s d = 0.62 vs 0.44).

**Table 2.** The demographic information of empathy scale scores (Jefferson Scale of Physician Empathy) N = 669

Variables	Mean	SD	Median	df	X ^ 2 ^	p-value
Year				4	19.62	0.001
1	108.09	11.25	110.0			
2	106.69	11.00	109.5			
3	102.41	12.36	104.0			
4	105.34	12.41	106.0			
5	104.60	11.71	105.0			
Gender				1	10.38	0.001
Male	104.3	11.78	105.0			
Female	106.9	11.59	109.0			
Age						
<22	106.5	11.99	108.0			
22-27	105.3	11.46	106.5			
>27	107.0	11.33	110.0			
Ethnicity				3	3.61	0.307
African	105.9	12.78	108.5			
Indian	106.3	10.88	107.0			
Mixed	106.0	12.42	107.0			
Other	99.0	12.90	101.0			
Specialization				1	0.09	0.765
People-oriented	106.2	11.42	107.0			
Technology-oriented	105.8	12.24	107.5			

### Toronto Empathy Questionnaire (TEQ)

The TEQ was successfully completed by 662 students with a Cronbach’s alpha of 0.85. Descriptive statistics are presented in Table 2 Similar to JSPE-S there was a significant effect for gender (X^2^(1)=25.27, p<0.001) with females’ mean TEQ scores being significantly higher than males’ (48.0 + 7.11 vs 45.3 + 7.24). There was also a significant effect of year of study (X^2^(4)=22.51, p<0.001). Post hoc analysis yielded a significant difference between mean empathy scores in years one and three (X^2^(1)=16.67, p<0.001) and years two and three (X^2^(1)=6.74, p=0.009). There was a medium effect size, Cohen’s d = 0.5. There were no significant effects of age, ethnicity or specialization, p>0.05, Table 2

### Reading the Mind in the Eyes Test (RMET)

This test was completed by 655 students. Descriptive statistics and testing for associations are found in Table 2 The overall trend was different from the other two tests with an increase in year two followed by a decrease in years three and four, Figure 1 There was no difference between year one and any of the other years, p>0.05. There was a significant effect of gender (X^2^(1)=14.59, p<0.001) with females again scoring higher than males (26.2 + 0.21 vs 24.7 + 0.30).

### Correlation Analysis

**Figure 2.**
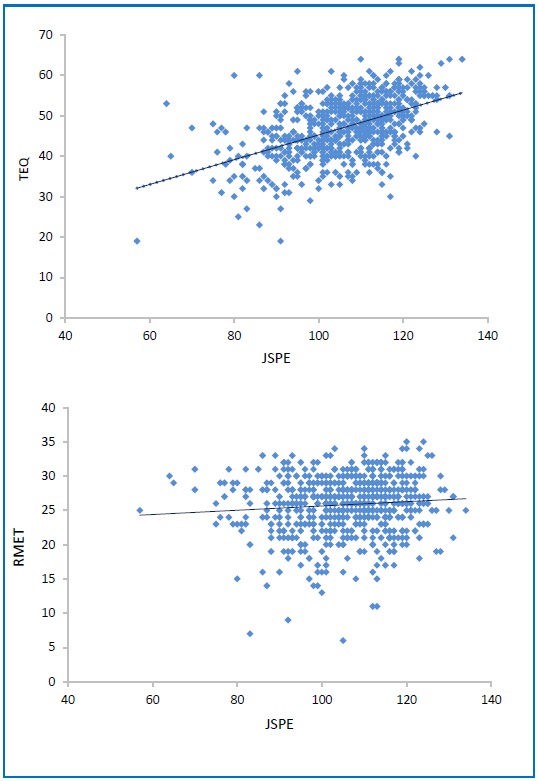
Correlation between the JSPE-S and the TEQ (A) and the JSPE-S and the RMET (B) empathy scales

Correlation analysis demonstrated a moderate and significant correlation between scores on the TEQ and the JSPE-S (ρ =0.48, p<0.001). However there was almost no correlation between the RMET and JSPE (ρ = 0.08, p=0.04), see Figure 2

**Table 3.** The demographic information of empathy scale scores (Toronto Empathy Questionnaire) N = 669

Variables	Mean	SD	Median	df	X^2^	p-value
Year				4	22.51	<0.001
1	48.6	11.25	49.0			
2	47.6	11.00	48.0			
3	44.8	12.36	44.0			
4	46.8	12.41	47.0			
5	45.9	11.71	46.0			
Gender				1	25.27	<0.001
Male	45.3	11.78	45.0			
Female	48.0	11.59	49.0			
Age				2	4.78	0.092
<22	47.6	11.99	48.0			
22-27	46.5	11.46	46.0			
>27	48.2	11.33	49.0			
Ethnicity				3	2.01	0.571
African	47.1	12.78	47.0			
Indian	46.8	10.88	47.0			
Mixed	47.7	12.42	49.0			
Other	46.3	12.90	45.0			
Specialization				1	0.89	0.316
People-oriented	47.6	11.42	47.0			
Technology-oriented	46.7	12.24	47.0			

**Figure 2** Correlation between the JSPE-S and the TEQ (A) and the JSPE-S and the RMET (B) empathy scales

**Table 4.** The demographic information of empathy scale scores (Reading the Mind in the Eyes Test) N = 669

Variables	Mean	SD	Median	df	X^2^	p-value
Year				4	14.27	0.006
1	25.9	4.3	27.0			
2	26.8	3.5	27.0			
3	25.3	4.8	26.0			
4	24.9	3.9	25.0			
5	25.7	4.2	26.5			
Gender				1	14.59	<0.001
Male	24.9	4.6	25.0			
Female	26.3	3.9	27.0			
Age				3	5.06	0.080
<22	26.1	4.18	27.0			
22-27	25.5	4.11	26.0			
>27	26.9	4.35	27.0	3		
Ethnicity					2.71	0.438
African	26.4	3.43	27.0			
Indian	25.6	4.53	26.0			
Mixed	26.1	3.94	27.0			
Other	26.3	3.64	26.5			
Specialization				1	0.83	0.361
People-oriented	25.7	4.16	26.0			
Technology-oriented	25.3	4.40	26.0			

## Discussion

This study provided a cross sectional empathy profile of medical students during their five years of training in Trinidad and Tobago. We report that final year students’ empathy scores on the JSPE-S were lower than those of first year students. While similar results have been reported in many studies^10^^, ^11^, ^22^, ^23 and our data demonstrates a medium effect size, Cohen’s d = 0.5, these findings are still considered controversial as one meta-analysis suggests.^24^ It is worth noting that most of the work reporting a decline in empathy has originated from medical schools in the United States, whereas reports originating in the United Kingdom appear to demonstrate no change or increases in empathy scores across medical training.^25^^, ^26 In developing nations, few studies have sought to examine this trend. A study done in Ethiopia showed an increase in cognitive empathy between the first and final year of training but showed no change in emotional empathy,.^21^ while a study conducted in Iran showed no difference throughout medical training.^27^

The difference between our results and those from the United Kingdom suggested further analysis of the declining trend was necessary as medical education in Trinidad and Tobago mirrors the five-year undergraduate program typical of most British Universities. This contrasts with the US system in which medicine is taught as a post-graduate degree over a four-year period. As such, it might have been expected that our results might more closely mirror our British counterparts. Yet this was not the case. Not only did final year students show significantly lower empathy scores but the downward trend in empathy was maximum during the third year of training before plateauing in years four and five. Our results thus mirror the pattern previously described in which researchers suggest that it is when students enter into their clinical years of training and are faced with the competing demands of studying and working with patients that this erosion of empathy begins to occur.^10^^, ^28

Previous work from our institution has noted a decline in empathy between entry into and completion of the first year of study, attributing this decline to a ‘settling in’ phenomenon and a shift from idealism to realism.^15^ We also report a difference in empathy scores between years one and two before a more substantial drop in year three. In their third year, students in Trinidad and Tobago transition from their preclinical studies to clinical years where they are exposed to actual patients. Factors thought to be responsible for this decline include continued loss of idealism, increased workload and a shortage of desirable role models. This last point is particularly important as the third year represents a transition in teaching methodology from the safety of the classroom to one where interaction with patients now becomes a critical model for knowledge acquisition. While the professor with PowerPoint slides typifies the early years of medical training and carries with this the familiar environment of high school, the clinical years are built around interactions with patients in clinics and hospitals that reflect the emotional roller coaster of real life.

In this clinical setting students are heavily influenced by their role models and the reality of the hidden curriculum as a primary determinant of behaviour sets in. Narrative reflections from third year students in the US highlight patient dehumanization, power hierarchy concerns in training, ‘hidden assessment’ of performance, suppression of normal emotional responses and pressure to ‘fake it’ as among the issues with which young ‘doctors’ are often forced to contend.^28^ These realities may be exacerbated in many developing countries such as Trinidad and Tobago, where clinicians often practice medicine with very few resources and thus have to contend with even more challenges.

Our second major finding was that measures of empathy as recorded by the JSPE-S mirrored almost exactly those from the second instrument we used to assess affective empathy, the TEQ, Figure 1 In fact there was a strong and significant correlation between the scores on both scales. The TEQ is a recently developed scale that measures empathy as primarily an emotional process. The TEQ demonstrated good internal reliability in our study and our data therefore suggests that empathy changes observed across years are not simply a result of perspective taking or cognitive empathy but also due to significant changes in affective empathy. It is likely that both the cognitive and emotional aspects of empathy are necessary and are unlikely to occur independent of each other. “Emotive attunement” helps guide the physician with regard to “when to ask questions, when to stay silent, and when to repeat important words” yet there can be no emotive attunement without first perceiving the patient’s point of view.

With respect to the RMET we did not find any difference in scores across year groups and this probably accounted for the fact that there was almost no correlation between the JSPE-S and the RMET. Importantly our scores are not only in line with previously published data but also concur with a more recent study conducted among medical students in Australia.^14^^, ^29 The study done in Australia also found no difference among different years of training; in this study there was also no difference in RMET scores between medical students and a group of older more experienced physicians who were also surveyed.

This lack of correlation between the JSPE-S and the RMET is somewhat surprising because as previously stated both scales are designed to measure cognitive empathy or ‘perspective taking’. One explanation for this discrepancy may be that the RMET only provides a narrow measure of cognitive empathy, i.e. the ability to simply recognise emotion in others. However JSPE-S in testing ‘perspective taking’ treats this ability to recognise emotions in others as inherent and therefore focuses on how that information is utilized as well as on the importance that is attached to that information. Another possible explanation for the differences between the scales is the potential response bias noted with self-report scales like the JSPE-S. More objective psychophysiological measures of empathy and emotional recognition tasks such as the RMET may provide better insight into determining the inherent empathetic capacity of individuals. The Australian study noted a ceiling effect when using the RMET. This may be a confounder in our study. They attributed this ceiling to the intelligence levels of the groups being tested though their results remain similar to ours, even after they attempted to negate this ceiling by limiting the time to recognise the emotion and including a distractor.

The use of more objective measurement tools and physiological and behavioural rather than self -reporting scales to assess empathy are clearly important. As demonstrated in the current study, a comparison of all three scales suggests that students show little difficulty in perceiving the emotional content of others across the five years of medical school (cognitive empathy). However, it is their affective response to these emotions that appears to change. Interestingly, a recent study demonstrated that physicians show decreased neuronal responses when observing others being exposed to painful stimuli.^30^ There was no doubt that the physicians were able to recognise the nature of the painful stimuli but the negative arousal associated with that observation was down-regulated. Recent research has also found that the stress associated with long shift hours also reduces empathetic responses, as does previous inexperience with painful states.^31^-^33^

These studies consequently provide a psychophysiological explanation for the decrease in self-report empathy scores seen in medical students. Blunting of the affective empathetic response does not necessarily represent a ‘hardening of the heart’ but rather suggests that the decline is due to underlying processes that might be necessary to preserve the finite resources available for cognitive processing. These responses may well be necessary to enable medical students to manage the often new and complex situations they face during their training.

The decrease in affective empathy and the shift to more cognitive processing is supported by a recent qualitative study done in Japan that explored how empathy is conceived among both medical students and residents.^34^ They also report that those further along in their clinical training demonstrate a decrease in affective empathy. However this study concludes that affective empathy in residents is replaced by a greater capacity to utilize cognitive empathy and thus suggest there is no overall decline. Indeed, it is essential that in sharing the emotions of others that medical students preserve their own emotional integrity in order to successfully treat their patients.

In conclusion, our data demonstrate that the empathy profile of students across the five years of medical training is variable with highest scores being observed when students enter medical school and lowest scores among third year students. This data is consistent with studies from other parts of the world and we therefore suggest that factors that have previously been cited as responsible for the decrease in empathy (including demanding work and examination schedules, dehumanization of patients and emotional distancing) are relevant and important in the Caribbean context. Indeed these factors can act as stressors and not only contribute to the decrease in empathy experienced by medical students, but also to their own individual well-being.

In this light our finding that there was no decline in cognitive empathy as measured by the RMET is important. It suggests that rather than failing to recognise the emotions being experienced by their patients, students may be demonstrating a reduced emotional response in an attempt to preserve cognitive processing capacity and thus manage the challenges they must negotiate in this new environment. Therefore, from a medical education perspective, administrators should ensure that students not only receive training pertaining to empathy but should also be provided with the means to improve their cognitive processing capacity in difficult circumstances. Training can include opportunities for self-reflection and discussion of the challenges students face with their patients, student access to support groups and counselling, as well as the development of educator mentoring processes.^35^ Moreover, such training can be more effective if it is rooted in the neurological underpinnings of emotion and empathy.^36^ Finally there is a need to develop other relevant psychophysiological and behavioural tools to measure empathy. These can provide deeper insight into the cognitive and emotional changes students undergo during their training and beyond.

### Limitations

Our study is limited by the fact that we present cross-sectional data. As such the changes in empathy reported do not reflect changes over time as would a longitudinal study but rather reflect differences between the various year cohorts. This prevents conclusive statements being made about a change in empathy levels throughout training within the Caribbean context. However, given that one of the main objectives of this study was to compare the use of various instruments and not measure trends over time, we believe the data provide insights into the level of empathy among the students that is consistent with the literature from other parts of the world. It also provides the backdrop for future longitudinal studies.

Another limitation is that our sample is drawn from one medical school and the majority of the participants (90%) were from one nation. While each Caribbean nation is unique in its own right, given common historical and socio-cultural patterns among the various islands we suggest that the data can also provide insight into the wider Caribbean experience. In addition participation in this study was voluntary which resulted in a disproportionately high percentage of year one students. The response rate for this group was 87% (vs 65% for the entire study) which represented approximately one third of the total sample. Perhaps this willingness to participate is a reflection of the idealism previously referred to that was associated with entry into medical school. The lowest response rate was seen in third and fourth year classes where just over half of the total class participated and was also consistent with the lower empathy scores on the JSPE and the TEQ. Finally, two of the instruments used were self-reporting scales. Therefore, it is not possible to assess to what extent students’ responses were based on their perception of social norms or their actual beliefs and whether these responses truly mirror behaviours. Our use of the RMET was meant to address this gap by providing a more physiological measure of empathy that operated independent of any social bias. A limitation of the RMET is that it uses fixed photographs of patients’ eyes, while in a real encounter there are more nonverbal cues available to help determine the underlying emotions experienced by a patient. As such it does not fully explore students’ ability to express cognitive empathy.
